# A dual task priming investigation of right hemisphere inhibition for people with left hemisphere lesions

**DOI:** 10.1186/1744-9081-8-14

**Published:** 2012-03-20

**Authors:** Erin R Smith-Conway, Helen J Chenery, Anthony J Angwin, David A Copland

**Affiliations:** 1 The University of Queensland Centre for Clinical Research, Brisbane, QLD, Australia; 2 The University of Queensland, School of Health and Rehabilitation Sciences, Brisbane, QLD, Australia; 3 The University of Queensland, Faculty of Health Sciences, Brisbane, QLD, Australia

## Abstract

**Background:**

During normal semantic processing, the left hemisphere (LH) is suggested to restrict right hemisphere (RH) performance via interhemispheric suppression. However, a lesion in the LH or the use of concurrent tasks to overload the LH's attentional resource balance has been reported to result in RH disinhibition with subsequent improvements in RH performance. The current study examines variations in RH semantic processing in the context of unilateral LH lesions and the manipulation of the interhemispheric processing resource balance, in order to explore the relevance of RH disinhibition to hemispheric contributions to semantic processing following a unilateral LH lesion.

**Methods:**

RH disinhibition was examined for nine participants with a single LH lesion and 13 matched controls using the dual task paradigm. Hemispheric performance on a divided visual field lexical decision semantic priming task was compared over three verbal memory load conditions, of zero-, two- and six-words. Related stimuli consisted of categorically related, associatively related, and categorically and associatively related prime-target pairs. Response time and accuracy data were recorded and analyzed using linear mixed model analysis, and planned contrasts were performed to compare priming effects in both visual fields, for each of the memory load conditions.

**Results:**

Control participants exhibited significant bilateral visual field priming for all related conditions (*p *< .05), and a LH advantage over all three memory load conditions. Participants with LH lesions exhibited an improvement in RH priming performance as memory load increased, with priming for the categorically related condition occurring only in the 2- and 6-word memory conditions. RH disinhibition was also reflected for the LH damage (LHD) group by the removal of the LH performance advantage following the introduction of the memory load conditions.

**Conclusions:**

The results from the control group are consistent with suggestions of an age related hemispheric asymmetry reduction and indicate that in healthy aging compensatory bilateral activation may reduce the impact of inhibition. In comparison, the results for the LHD group indicate that following a LH lesion RH semantic processing can be manipulated and enhanced by the introduction of a verbal memory task designed to engage LH resources and allow disinhibition of RH processing.

## Background

Language processing is dynamic and requires the participation of both cerebral hemispheres. The left hemisphere (LH) is considered to be the language dominant hemisphere, however, the right hemisphere (RH) is also accepted to play an important role in language processing. The RH has been linked with processing of discourse, comprehension of inferences, ambiguity and metaphoric language, and underlying much of this, is its role in lexical-semantic processing [see for a review of RH language processing [1]]. According to dynamic models of cognitive functioning bilateral lexical-semantic processing will involve both interhemispheric activation and inhibition [2]. The language dominant LH is suggested to inhibit aspects of RH participation in order to maximize the efficiency of word level processing and meaning selection [2-4]. Interhemispheric inhibition has been suggested to limit the RH's ability to perform to its maximum semantic processing capacity under normal processing conditions, and to therefore have led to an underestimation of the RH's semantic processing ability [5].

Conditions that overload the LH's control mechanisms and facilitate the RH's release from inhibition are suggested to allow the RH to exceed its traditional performance on word level lexical-semantic tasks [6]. There is, therefore, the potential for RH disinhibition to play a role in the increases in RH activity observed in neuroimaging investigations of neural reorganization [7-12] and improvements found for behavioral online RH semantic processing performance [13-15] following LH lesions. However, investigations of hemispheric contributions to semantic processing following unilateral lesions have not directly measured the impact of RH disinhibition or residual LH suppression on the performance of each hemisphere. Therefore, the current study will reexamine hemispheric contributions to semantic processing following LH lesions under conditions designed to manipulate the degree of interhemispheric activation and inhibition.

Existing theoretical insights into hemispheric performance during semantic processing in healthy adults under normal processing conditions (i.e., without manipulation of RH disinhibition) suggest that both hemispheres are capable of processing lexical-semantic information, and that hemispheric differences relate to an interaction between the strength of relatedness of items and the time-course allowed for semantic access [16]. This time-course hypothesis suggests that activation in the LH begins broadly and includes both strongly and weakly related items, followed quickly by the focusing of activation to include closely related items only. Alternatively, the RH is slow to initiate, but once activation is achieved, the RH will maintain that activation for both closely and distantly related items over time [16]. Accordingly, the RH's role in semantic processing appears to be to maintain activation of multiple interpretations that can be called upon by the LH when complex meaning integration is required [17].

Theories of hemispheric semantic activation are based predominately on findings of divided visual field (DVF) semantic priming investigations. Semantic priming refers to the facilitation effect wherein a target word is responded to more quickly and accurately when it is preceded by a related word (prime), compared to an unrelated one. Semantic priming is therefore a measure of the spread of activation in the semantic system. The DVF methodology is used in order to assess activation in each hemisphere separately, via the presentation of prime and/or target letter strings to the left visual field (LVF) or right visual field (RVF). Prime and target pairs related via category membership and/or association have been one focus for investigations into the time-course of hemispheric activation. These stimuli represent a hierarchy of activation that can differentiate hemispheric patterns of activation with respect to the interaction between relationship strength and time-course of semantic activation [e.g., [16,18]]. Particular patterns of activation are suggested for each relationship type.

Items that are related via both category membership and association (e.g., DOG--CAT) are suggested to be the most strongly related pair type, compared to those related via category membership or association alone. This is due to the additive nature of the semantic (category membership) and lexical (association) relationships, and because these items are suggested to share the most semantic features [e.g., [19]]. Evidence is accumulating that items related by category membership and association generally exhibit bilateral activation over time [18-20].

Items related via association but not category membership (e.g., DOCTOR--HOSPITAL) are considered the next most strongly activated due to the benefit of the associative relationship [21]. Behavioral and event-related potential priming studies have found predominately LH advantage for priming association only pairs, prior to 500 ms stimulus onset asynchrony (SOA; time between onset of prime and target) [22-24]. There have been some suggestions that the RH is capable of priming this pair type at longer SOAs, in accordance with the time-course hypothesis [22].

Finally, items that are related via category membership but not association (e.g., CAMEL--DOG) represent the weakest relationship. These items may share some semantic features but do not benefit from the associative boost, and often exhibit the weakest priming effect [21]. Investigations of items related via category membership only suggest that the variation in hemispheric processing patterns relate to underlying processing conditions, with a LH asymmetry under automatic spreading activation conditions [25-27] and a RH asymmetry under conditions that encourage strategic processing [26-28] or at long SOAs [16].

DVF priming investigations have provided insight into the semantic processing of the healthy brain under normal processing conditions. However, the RH disinhibition hypothesis suggests that RH performance described in such studies does not reflect its true capacity [5]. Specifically, the RH disinhibition hypothesis suggests that the RH is less active and does not perform to capacity when normal LH dominance remains, however when this balance is modified the RH is able to demonstrate a greater degree of semantic processing e.g., [29]. Supporting evidence for the RH disinhibition hypothesis has been derived from investigations of people with LH lesions [30,31] and investigations that attempt to release the RH from LH suppression via experimental manipulations, such as short presentation times for lateralized stimuli [30], incorporation of a secondary task to "overload" LH processing [5,6,29], or simulation of lesions using rTMS [11]. These investigations have found it possible to release the RH from unilateral suppression by manipulating the attentional balance between the hemispheres.

Manipulation of the interhemispheric attentional balance has been most specifically assessed using a combination of the dual task and DVF paradigms with healthy participants. The dual task paradigm takes advantage of functional task lateralization in order to either pre-activate or overload the attentional resources of a particular hemisphere. The concurrence decrement effect, that occurs when a particular hemisphere is overloaded, can be explained using the multiple limited resources model [32]. The multiple limited resource model dictates that each hemisphere has a finite supply of attentional resources which cannot be shared. Therefore, when two sufficiently difficult concurrent tasks are performed by a particular hemisphere, the resource demands overlap to such an extent that the hemisphere becomes overloaded. Dual task investigations of semantic processing have used secondary verbal memory tasks in order to overload the LH's resources and produce a RH disinhibition effect and subsequent improvements in RH processing [e.g., [5,6,29]]. The combination of DVF presentation in a dual task experiment allows the direct investigation of individual hemispheric performance following disinhibition.

DVF dual task investigations have found improvements in RH processing following disinhibition. Specifically these investigations have reported that RH disinhibition results in the reduction of the traditional LH processing advantage for lexical decision tasks, and RH activation of items traditionally associated with LH processing, such as long low imageable words [5,6]. Similarly, participants with partial disconnection between the hemispheres have also exhibited an enhanced RH semantic processing performance following RH disinhibition under dual task conditions [29].

The findings from DVF dual task investigations [e.g., [5,6]], and in turn the RH disinhibition hypothesis, have interesting implications for the study of changes in hemispheric contributions to semantic processing for people who have suffered lesions to the language dominant LH. For instance, behavioral investigations of hemispheric contributions to semantic processing for people with LH lesions and subsequent aphasia, have found evidence of improved RH lexical-semantic abilities following LH lesions [13-15] and neuroimaging investigations have found increases in RH activation during language tasks following LH lesions [7-12]. Furthermore, investigations of spatial attention treatment for people with LH lesions have explored the effect of manipulating the participation of each hemisphere, and have found that priming or recruiting attentional or intentional mechanisms in the intact RH (via orienting attention to the hemispace or a complex left handed movement) can improve language function in some people with aphasia [33-35]. However, the potential influence of RH disinhibition is yet to be fully explored in the context of the individual hemispheric contributions to language processing following LH lesions.

The current investigation seeks to extend the examination of changes to RH language function post LH lesion, in order to more directly explore the impact of the lesion on RH disinhibition and to explore the impact of RH disinhibition on RH performance. Therefore the current investigation will compare hemispheric performance on a DVF semantic priming task (primary task) over three verbal memory load conditions (concurrent task). Semantic priming effects may arise from one of three mechanisms; automatic spreading activation, controlled expectancy-based activation and controlled post-lexical semantic matching [36]. The current investigation will encourage controlled activation by using a high relatedness proportion (ratio of related word targets and unrelated word targets; the higher the proportion the more controlled) [36], as hemispheric differences appear to occur under controlled processing conditions [16,37]. A shorter SOA (250 ms) was employed to allow post-lexical controlled processing, as this onset asynchrony is insufficient for the generation of expectancies. Post-lexical semantic matching occurs after the presentation of the target and before the lexical decision is made [36] and involves checking from target back to prime, to assess semantic relatedness. The presence of a semantic relationship indicates that the target is a real word and therefore accelerates a word response [38].

The stimuli conditions (category and association, category only and association only items) chosen for the current primary semantic priming task were included because of their sensitivity to hemispheric priming differences [e.g., [19,22,25,39]]. In particular, the association only condition is hypothesized to provide a platform for the RH to exhibit an increase in priming performance, with previous findings suggesting that the RH exhibits some difficulty processing items related via association only in healthy adults at shorter SOAs [22]. However, given that both hemispheres have access to the same semantic network, following disinhibition the RH will become more active and have the potential to activate all of the related conditions. It is also noted that association only stimuli have been found to prime during central priming investigations of people following LH lesion [e.g., [40]], therefore it is plausible that the RH is capable of activating these stimuli under conditions that promote RH performance.

It is hypothesized that a different pattern of priming is expected for each of the related stimuli conditions depending on the degree to which RH disinhibition is occurring. The related conditions are expected, under normal processing conditions (ie. no disinhibition), to exhibit bilateral priming for items related by category membership and association, priming in the LH for the association only condition and priming in the RH for the category only condition, as well as exhibiting an overall LH response time advantage. Under conditions that elicit RH disinhibition it is expected that RH priming will increase to include significant priming of the association only condition, and that the overall LH processing advantage will be removed.

For control participants it is expected that the 6-word concurrent memory task will sufficiently modify the attentional balance between the hemispheres, and in accordance with the RH disinhibition hypothesis, result in an increase in RH priming. It is hypothesized that performance by participants with a LH lesion will differ from the control participants, as they will be more susceptible to the demands of the concurrent task, and to the impact of the lesion itself on hemispheric control. If the LH lesion results in disinhibition of RH semantic processing, then participants with a LH lesion should exhibit similar activation during both baseline and memory load conditions, including priming of all three related conditions in the RH. Conversely, if the LH lesion does not result in complete RH disinhibition, then improvement in RH processing is expected to improve under the 2- word and 6-word conditions, with priming of association only stimuli occurring in the RH when the concurrent load is sufficient to overload LH resources.

## Methods

### Participants

Participation in this experiment was voluntary and written informed consent was obtained from all participants prior to participation. The research was approved by the Medical Research Ethics Committee at The University of Queensland, Brisbane, Australia.

Nine people with a single LH lesion (3 males, 6 females) and 13 healthy matched control participants (3 males, 10 females) took part in this investigation. Participants in the LH damage (LHD) group exhibited impaired language abilities compared to controls, with a significant difference between groups for the Western Aphasia Battery (WAB) [41] aphasia quotient, *t*(20) = -5.356, *p *= .002, and with each participant in the LHD group scoring more than one standard deviation below the control group mean aphasia quotient (M = 99.46; SD = .7974). A summary of the LHD group participant information is included in Table 1.

**Table 1:** Summary of Participants in the LHD group

*Case*	*Age*	*Gender*	*Education* *(years)*	*Lesion site*	*Months post stroke*	*AQ*	*WAB classification*	*CLQT*
1	71	M	7	Occipital	36	98.4	Nonaphasic	4.0

3	63	F	17	Parietal	180	98.4	Nonaphasic	4.0

11	60	F	10	Temporal-Parietal	18	91.4	Unclassified residual	4.0

13	39	F	12	Parietal & Striato capsular	82	95.8	Nonaphasic	4.0

14	49	F	19	Fronto-Tempo-Parietal	13	89.9	Anomic	3.8

20	77	F	7	Parietal	68	97.6	Nonaphasic	4.0

21	58	M	16	Fronto-Parietal	57	93	Unclassified residual	3.8

23	76	M	9	Parietal	10	91.3	Anomic	4.0

58	64	F	15	MCA territory	50	89.7	Anomic	3.8

*Abbreviations: LHD *Left hemisphere damage, *AQ *Western Aphasia Battery Aphasia Quotient, *WAB *Western Aphasia Battery, *CLQT *Cognitive Linguistic Quick Test overall score, *MCA *middle cerebral artery

Participants in the LHD group had a mean age of 61.7 years (range 39-77; SD 12.3) and a mean of 12.4 years of education (range 7-19; SD 4.48). The control group had a mean age of 60.7 years (range 37-77, SD 10.77), and a mean of 15 years of education (range 8-25, SD 4.89). *t*-tests comparing the LHD and control groups revealed no significant difference with respect to age, *t*(19) = .011, *p *= .991 (two-tailed), or education, *t*(17) = -.963, *p *= .349 (two-tailed). Participants in the LHD group met the following criteria (a) pre-morbidly right handed; (b) native English speaker; (c) no reported history of traumatic brain injury, dementia, brain tumor, aneurisms, cerebral abscess or alcoholism; (d) scored within normal limits (> 3.5) on the Cognitive Linguistic Quick Test [42] composite severity rating; (e) no visual field deficit or other uncorrected visual or hearing deficit; and (f) at least six months post lesion at the time of testing. Control participants were right handed, native English speakers, and were excluded if (a) they had reported history of neurological impairment, brain injury or alcoholism; or (b) had uncorrected visual or hearing impairments.

### Stimuli and design

The experiment was a 2 × 2 × 4 × 3 (Group × Target Visual Field × Stimuli Condition × Memory Load) mixed factor with group (control, LHD) as a between-subjects factor, and target visual field (LVF and RVF), stimuli condition (Category and Association, Category Only, Association Only, Unrelated) and memory load (0-, 2-, and 6-word) as within-subject factors. The stimuli employed in the lexical decision priming task (LDT) (primary task) consisted of 320 visually presented prime-target pairs. All primes were nouns, and targets were either real word nouns or legally spelled non-words. The ratio of real word to non-word targets was 1:1, in order to negate response bias. Critical pairs (with real word targets), comprised four pair types. Category and association (CA) prime-target pairs are related via category membership [43,44] and are listed as primary associates [45-50] (e.g., cat-dog). Category only (CO) pairs are related via category membership [43,44], and are not listed as associates (e.g., deer-pig). Association only (AO) pairs are listed as primary associates in association norms [45-50], and are not categorically related (e.g., bee-honey). Finally, unrelated (UNR) pairs were formed with words that are not related via category membership or association. The relatedness proportion was 0.75 (number of related word targets/total number of word targets).

Primes and targets were three to six letters in length. Stimuli were matched between the related conditions and between the related and unrelated conditions, with no significant difference (*p *> .1) found for the variables of word length, frequency [51] or imageability [52-54]. Conditional means for each of these stimuli features are presented in Table 2. Targets were also matched across stimuli types for lexical decision response time (RT); each target's average RT (when presented for single word lexical decision) was accessed from the English Lexical Project website [55] and matched to ensure that any RT differences are due to priming effects and not underlying differences in lexical decision time. Non-word and real word targets were matched for word length, *t*(318) = -.603, *p *= .547 (two-tailed).

**Table 2:** Summary of Stimuli Features

*Stimuli Type*	*Number of Letters*		***Frequency***^***a***^		*Imageability^b^*		*LD RT (ms)^c^*	
	
	*Prime*	*Target*	*Prime*	*Target*	*Prime*	*Target*	*Prime*	*Target*
Category and Association	4.8	4.4	67.3	61.8	594.6	594.4	602.1	598.6

Category Only	4.5	4.5	48.5	80.2	583.8	589.6	599.8	608.9

Association Only	4.5	4.5	49.4	86.4	585.7	582.7	609.9	596.2

Unrelated	4.8	4.5	66.3	82.2	587.1	588.5	603.6	599.8

Mean number of letters, mean frequency, mean imageablity and mean lexical decision response time for the prime and target items in each condition. *t*-tests between each condition for each feature reveal no significant differences (*p *> .1). *Abbreviations LD RT *Single word lexical decision response time in milliseconds; ^a ^= Sourced from Kucera & Francis, 1967 [51]; ^b ^= Sourced from Gilhooly & Logie, 1980, Pavio, Yuille & Madigan, 1968, and Toglia & Battig, 1978 [52-54]; ^c ^= Sourced from The English Lexicon Project website [55]

Primes were centrally presented. Targets were presented at 2° of horizontal visual angle from central fixation. Half of the targets were presented to the left visual field (LVF) and half to the right visual field (RVF). The total list of 320 pairs seen by participants is made up of 80 real word target pairs (20 in each related condition and 20 unrelated) and 80 non-word target pairs, and in order for each of the target items to be presented to both visual fields over the course of the experiment, pairs are repeated in a mirror image list [as per [18,19,27,37,39,56]]. The order of initial visual field of presentation for each target was counterbalanced between participants. The same stimuli were used for the primary priming task in each of the three memory condition experiments, presented in three separate sessions.

The verbal memory stimuli and distracter items employed in the secondary verbal memory task included words with low frequency and low imageability, in order to target LH processing. Stimuli items were between 3 and 13 letters in length ($\bar{x}=\text{7}$; SD 2.5), had a mean frequency of 19.2 (range 1-91; SD 20.3) [51] and a mean imageability of 382.5 (range 216-499; SD 58.5) [52-54]. No memory or recognition items were repeated within a session.

The practice list for the zero word memory session incorporated 10 word and 10 non-word targets for the LDT. The practice lists for the two and six word memory sessions included both the memorization and LDT stages. The practice tasks did not include any items used in the actual experiment.

### Procedure

Participants were seated 60 cm from the laptop computer monitor, and provided with a chin rest to control head movement and assist with maintenance of central fixation. All stimuli were written in black 24 point courier new font lowercase letters, presented on a white background. Stimuli presentation, timing and data collection was controlled by E-Prime experimental software version 1.1 (Psychology Software Tools), and responses were recorded via the PST serial response box (Psychology Software Tools).

Each participant took part in three sessions, in order to complete each of the memory conditions; zero, two and six words. The sessions were separated by at least two weeks and session order was counterbalanced between participants. The first session began with an additional DVF LDT, designed to determine the optimum target duration time for each participant [14]. Ten trials (none of which were included in the experiment) were presented, half to each visual field, with the participant asked to make a lexical decision for each trial, while maintaining central fixation. The timing of target presentation was modified according to the individual's accuracy and eye movement. The first run of 10 trials was conducted at 180 ms target duration, followed by subsequent runs of trials (different stimuli each run) at durations increasing by 50 ms each run (e.g., 180, 230, 280 ms etc.), until the participant achieved the pass score of 70% correct on trials with no eye movement.

After determining optimum target duration, the participant would begin with the practice block. Experimental trials for the zero word condition were split into eight blocks of 40 LDT priming trials (described below). Participants were encouraged to take a short break between blocks. The two- and six-word memory condition trials were also split into eight blocks; each block included five segments, and each segment was made up of the following three stages:

#### Stage 1- Memorization

Memory lists were either two or six words in length. Each memory word was centrally presented, one-at-a-time, for 1000 ms. The list was repeated, so that each participant saw each word twice. Participants were instructed to read the words silently and memorize them for a subsequent recognition task.

#### Stage 2- LDT

Eight priming trials were presented per segment. Trials were pseudo-randomized to ensure that a single target type or visual field was not sequentially presented for more than three trials. Each trial began with the presentation of the central fixation cross, which remained throughout the entire trial, and after 2000 ms the central prime was presented one line above fixation for 150 ms. Prime and target presentation was separated by 100 ms (250 ms SOA). Targets appeared in-line with central fixation (height of 1.4^○ ^of a visual angle), pseudo-randomly to the LVF or RVF, stimuli subtended 2^○ ^(inner edge)- up to 7.6^○ ^(outer edge) of visual angle horizontally from the centre point. Targets were presented for between 180 ms and 380 ms, as determined by the individualized assessment of optimal target duration. The LHD group had a mean target duration of 327 ms (range 230-430 ms), and the control group had a mean target duration of 251 ms (range 180-330 ms). Following target presentation the fixation cross remained on screen for 5000 ms, to allow time for a response. Participants were instructed to record their lexical decisions by pressing one of two response buttons, to indicate a word or non-word response. Accuracy and RT were recorded and stored for analysis. Participants responded with their current dominant hand. Participants were instructed to maintain central fixation throughout the trials and eye movement was recorded online by the investigator; a video camera was trained on the participant's eyes and trials with eye movement were recorded and later discarded from analysis.

#### Stage 3- Recognition of Memorized list

Following the LDT, participants were presented with a four (two word memory condition) or a 10 (six word memory condition) word recognition list, presented one word at a time. The recognition items were centrally presented and participants were asked to indicate whether the item had been part of the memory list. The recognition words remained on screen until a response was made by pressing yes/no response buttons. The recognition lists were made up of all of the items presented in the memorization stage plus novel distracter items, inserted randomly within the presentation order. The recognition task was included to ensure the participants maintained memorization throughout the LDT. Accuracy was recorded for analysis.

### Statistical analysis

Analyses were conducted using Predictive Analytic SoftWare (PASW Statistics 18). Statistical analyses were carried out on RT and accuracy data corresponding to real word targets that were free from eye movement. Group differences in semantic priming effects (for RT and accuracy) were analyzed using Linear Mixed Model (LMM) analysis, with group (LHD, Control) as a between-subjects factor, and target visual field (LVF, RVF), stimuli condition (CA, CO, AO, UNR) and memory load (0-, 2-, 6-word) as fixed factors. Target duration and target repetition were treated as covariates. Data were then split according to memory load, in order to explore the effects of the secondary memory task on the primary LDT, and analyzed using LMM with group (LHD, Control) as a between-subjects factor, and target visual field (LVF, RVF) and stimuli condition (CA, CO, AO, UNR) as fixed factors. Target duration and target repetition were treated as covariates. LMM is used in order to account for variance between items, as well as between participants [57]. Pearson correlations were carried out between accuracy scores from memory recognition task and the accuracy scores from the LDT. The alpha level for statistical significance was set at 0.05 throughout.

## Results

### Response time analysis

Trials were excluded from RT analysis if responses were incorrect (LHD: 19.7%; Control: 10.8%), if eye movement was observed (LHD: 14.6%; Control: 12.4%), if RT deviated from the individuals conditional mean by more than two standard deviations, and if RT was extreme (> 2000 ms) and remained following removal of outliers, (LHD: 11.3%; Control: 5.5% of correct eye movement free trials). As the data violated assumptions of normal distribution, a logarithmic transformation was employed. All data analysis was then carried out on the transformed data; however, for ease of comprehension, raw data will be reported for descriptive purposes. Table 3 lists the group mean RT data in each condition for each memory load.

**Table 3:** Estimated Marginal Mean RT for LHD group and Control group participants

*Condition*	*0 word memory*				*2 word memory*				*6 word memory*			
	
	*LHD*		*Control*		*LHD*		*Control*		*LHD*		*Control*	
	
	*LVF*	*RVF*	*LVF*	*RVF*	*LVF*	*RVF*	*LVF*	*RVF*	*LVF*	*RVF*	*LVF*	*RVF*
CA	958.61	913.44	691.78	667.34	940.44	955.35	710.29	698.88	919.31	932.00	708.04	696.42
	
	(74.79)	(75.21)	(59.65)	(59.69)	(79.24)	(79.59)	(63.39)	(63.30)	(78.72)	(79.00)	(63.04)	(63.04)

CO	1050.75	959.56	778.73	730.75	1094.82	1023.89	783.86	762.41	1040.49	944.13	794.14	765.52
	
	(75.37)	(75.22)	(59.88)	(59.71)	(79.35)	(79.86)	(63.60)	(63.40)	(78.97)	(79.23)	(63.29)	(63.05)

AO	964.22	962.06	715.62	754.11	1019.91	1005.18	744.52	736.34	985.17	959.19	747.55	749.65
	
	(75.20)	(75.20)	(59.85)	(59.67)	(79.38)	(79.49)	(63.60)	(63.39)	(79.07)	(78.99)	(63.29)	(63.03)

UNR	1090.40	1058.32	872.30	826.94	1166.77	1103.68	846.83	855.92	1095.71	1062.84	862.42	872.90
	
	(75.36)	(75.84)	(60.08)	(59.97)	(79.69)	(80.44)	(63.73)	(63.56)	(79.43)	(79.47)	(63.62)	(63.29)

Estimated marginal mean response times in milliseconds with standard error in parentheses. Covariates for the estimated marginal means are Target Duration and Repetition (as per LMM analysis). *Abbreviations: CA *Category Association, *CO *Category Only, *AO *Association Only, *UNR *Unrelated, *RT *reaction time, *LHD *Left Hemisphere Damage group, *Control *Control group, *LVF *Left visual field, *RVF *Right visual field

The LMM revealed significant main effects for group [*F *(1,21) = 7.564, *p *= .012], memory load [*F *(2, 7464) = 9.549, *p *< .001], stimuli condition [*F *(3, 7464) = 216.500, *p *< .001], and target visual field [*F *(1, 7464) = 14.642, *p *< .001], and significant interactions between group and memory load[*F *(2, 7464) = 5.478, *p *= .004] and between target visual field and stimuli condition [*F *(3, 7464) = 4.316, *p *= .005]. The group main effect reflected that overall, the control group (767 ms) was significantly faster than the LHD group (1013 ms). The target visual field main effect reflected the overall RVF RT (879 ms) advantage over the LVF (901 ms). The memory load main effect reflected that when groups were combined the 0-word condition (876 ms) was significantly faster than both the 2-word (904 ms) [*p *< .001] and the 6-word (890 ms) [*p *= .007] conditions, with no difference between the 2- and 6-word conditions [*p *= .711]. When the groups were separated (group × memory load interaction) the control group was found to be significantly faster in the 0-word (751 ms) than both the 2-word (769 ms)[*p *= .002] and 6-word (780 ms) [*p *< .001] memory load conditions, with no difference between the 2- and 6- word conditions [*p *= .772]. The LHD group exhibited a different pattern with the 2-word condition (1038 ms) being significantly slower than both the 0-word (1002 ms) [*p *= .030] and 6-word (999 ms) [*p *= .037] conditions, which did not differ from each other [*p *> .1]. The target visual field and condition interaction reflected a significant advantage for RVF/LH RT (874 ms) compared to LVF/RH (924 ms) for the CO condition [*p *< .001], with no hemispheric differences for either the CA or AO conditions [*p *> .1].

### Semantic priming effects by memory load

**Figure 1:**
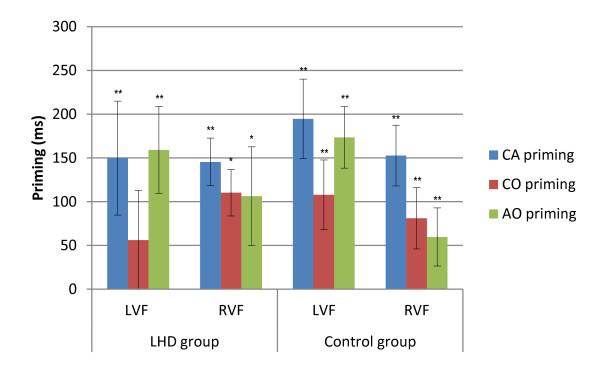
**Reaction time priming effects for LHD and Control groups in the zero-word memory condition**. Illustrated priming effects represent the mean of individual priming effects in milliseconds (Unrelated - Related condition RT) for each condition. Error bars represent +/- 1 Standard Error. LHD = Left hemisphere damage group; Control = Control group; CA = Category Association; CO = Category only; AO = Association only; UNR = Unrelated; LVF = Left visual field; RVF = Right visual field; * *p *≤ .05; ** *p *≤ .001.

The transformed RT data from the zero-word memory condition were subject to the LMM analysis described above, and revealed significant main effects for group [*F *(1, 21) = 7.805, *p *= 0.11], stimuli condition [*F *(3, 2526) = 67.229, *p *< .001], and target visual field [*F *(1, 2527) = 9.660, *p *= .002], and a significant interaction between target visual field and stimuli condition [*F *(3, 2526) = 4.901, *p *= .002]. The group main effect reflected the control groups RT advantage (755 ms) over the LHD group (995 ms). The target visual field main effect reflected a RT advantage for the RVF/LH (859 ms) compared to the LVF/RH (890 ms). Planned contrasts of hemispheric advantage were also carried out for each group separately, in order to assess changes in hemispheric dominance between memory load conditions. The LHD group comparison revealed a RVF/LH (973 ms) advantage, compared to the LVF/RH (1015 ms) [*p *= .02], and the control group comparison revealed no significant difference between the visual fields (LVF: 765 ms; RVF: 745 ms) [*p *= .118]. Conditional differences in each visual field were explored via planned contrasts for both groups. The LHD group exhibited bilateral priming for the CA [LVF: *p *< .001; RVF: *p *< .001] and AO [LVF: *p *< .001; RVF: *p *= .005] conditions, with CO priming reaching significance in the RVF only [*p *= .003]. The control group exhibited significant bilateral priming for all related conditions [*p *< . 001]. The priming effects (difference between means: UNR condition - related condition) for each group are presented in Figure 1.

In order to further explore differences in priming magnitude (UNR RT - Related RT) between the conditions and visual fields, a series of related sample t-tests were carried out. The comparisons for the LHD group revealed that the CA (135 ms) [*t *(8) = 3.524, *p *= .008] and AO (115 ms) [*t*(8) = -2.579, *p *= .021] conditions had a significantly larger priming effect than the CO condition (25 ms) for LVF targets. There were no significant differences between priming effects in the LH for the LHD group. Contrasts for the control group similarly revealed that LVF/RH priming effects for the CA (193 ms) [*t*(13) = 6.439, *p *< .001] and AO (168 ms) [*t*(13) = -4.162, *p *= .001] conditions were significantly larger than the CO condition priming effect (104 ms). In the RVF/LH, control participants exhibited a significantly larger priming effect for the CA condition (168 ms) compared to both the CO (99 ms) [*t*(13) = 5.110, *p *< .001] and the AO (71 ms) [*t*(13) = -4.610, *p *< .001] conditions. Comparisons between visual fields revealed a significantly larger priming effect for the AO condition in the LVF/RH (168 ms), compared to the RVF/LH (71 ms) [*t*(13) = 3.724, *p *= .003]. No other contrasts reached significance.

**Figure 2:**
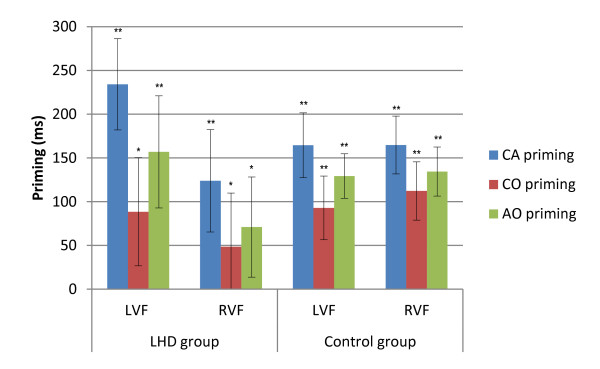
**Reaction time priming effects for LHD and Control groups in the two-word memory condition**. Illustrated priming effects represent the mean of individual priming effects in milliseconds (Unrelated - Related condition RT) for each condition. Error bars represent +/- 1 Standard Error. LHD = Left hemisphere damage group; Control = Control group; CA = Category Association; CO = Category Only; AO = Association Only; UNR = Unrelated; LVF = Left visual field; RVF = Right visual field; SOA = stimulus onset asynchrony; * p ≤ .05; ** *p *≤ .001.

The LMM for the two-word memory condition revealed significant main effects for group [*F *(1, 21) = 7.847, *p *= 0.11], stimuli condition [*F *(3, 2434) = 80.197, *p *< .001], and target visual field [*F *(1, 2435) = 5.062, *p *= .025]. The group main effect reflected the control group's RT advantage (767 ms) compared to the LHD group (1039 ms). The target visual field main effect reflected the pattern of RVF targets (893 ms) being responded to significantly faster than LVF targets (913 ms). Planned contrasts of hemispheric advantage were also carried out for each group separately, and revealed no significant difference between the visual fields for either group (LHD: LVF 1055 ms, RVF 1022 ms; Control: LVF 771 ms, RVF 763 ms) [LHD *p *= .057; Control: *p *= .293]. The interactions did not reach significance. The lack of interaction between group and stimuli condition suggests no group differences in the pattern of priming effects at the two word memory condition. Planned contrasts between stimuli conditions found that both groups exhibited bilateral priming for all related conditions [*p *< .01] as illustrated in Figure 2.

Priming magnitude was also compared for each group using related *t*-tests in the two-word memory condition. Contrasts for the LHD group revealed that for LVF/RH targets the CA condition had a significantly greater priming effect (214 ms) than the CO condition (61 ms) [*t*(8) = 5.361. *p *= .001] and the AO condition (141 ms) [*t*(8) = -2.662. *p *= .029]. In the RVF/LH the CA condition (146 ms) was found to have a significantly larger priming magnitude than the CO condition (85 ms) [*t*(7) = 3.498, *p *= .010]. Comparisons between visual fields revealed an advantage for CA priming magnitude in the LVF (214 ms) compared to the RVF (146 ms) [*t*(8) = 2.667, *p *= .032] for the LHD group. Control group comparisons revealed a significant difference between the priming magnitude for the CA condition (145 ms) and the CO condition (69 ms) in the LVF/RH [*t*(13) = 4.145, *p *= .001]. Similarly in the RVF/LH the CA condition (158 ms) had a significantly larger priming magnitude than the CO condition (94 ms) [*t*(13) = 3.625, *p *= .003], and the AO condition (110 ms) [*t*(13) = -2.19, *p = *.047].

**Figure 3:**
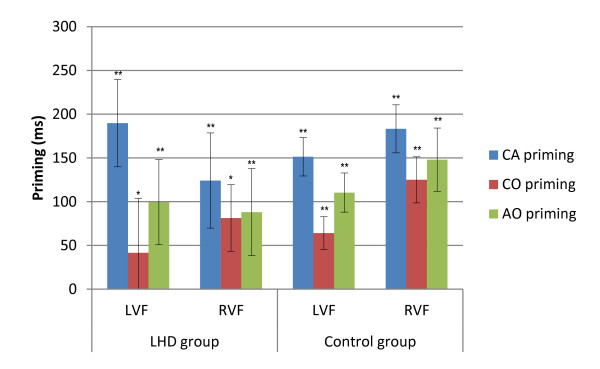
**Reaction time priming effects for LHD and Control groups in the six-word memory condition**. Illustrated priming effects represent the mean of individual priming effects in milliseconds (Unrelated - Related condition RT) for each condition. Error bars represent +/- 1 Standard Error. LHD = Left hemisphere damage group; Control = Control group; CA = Category Association; CO = Category Only; AO = Association Only; UNR = Unrelated; LVF = Left visual field; RVF = Right visual field; SOA = stimulus onset asynchrony; * *p *≤ .05; ** *p *≤ .001.

The LMM for the six-word memory condition data revealed significant main effects for group [*F *(1, 21) = 5.902, *p = *.024], and stimuli condition [*F *(3, 2462) = 86.426, *p *< .001]. The interactions did not reach significance in the six word memory condition. The group main effect reflected that the control group was significantly faster at responding (775 ms) than the LHD group (999 ms). Planned contrasts between stimuli conditions were carried out, and revealed significant bilateral priming for each of the related conditions [*p *< .05] for both groups; priming effects are illustrated in Figure 3. Planned contrasts of hemispheric advantage were also carried out for each group separately, and revealed no significant difference between the visual fields for either group (LHD: LVF 1010 ms, RVF 987 ms; Control: LVF 778 ms, RVF 771 ms) [LHD: *p *= .453; Control: *p *= .374].

Contrasts exploring differences in priming magnitude revealed a significant difference in priming effect between the CA (240 ms) and the CO (110 ms) conditions in the LVF for the LHD group [*t*(7) = 2.719, *p *= .030]. The control group comparisons revealed that the CA condition (170 ms) had a significantly larger priming effect than both the CO (77 ms) [*t*(12) = 4.555, *p *= .001] and AO (129 ms) [*t*(12) = -3.471, *p *= .005] conditions in the LVF, and a greater priming effect for the CA (204 ms) condition compared to the CO (132 ms) in the RVF [*t*(13) = 3.249, *p *= .006]. The between visual field comparisons did not reach significance.

Priming magnitude was also compared between memory loads for both groups. Each stimuli condition in each visual field was compared between zero- and two-word memory load conditions, between two- and six-word memory load conditions and between zero-word and six-word memory load conditions. None of the comparisons reached significance for either the control group or the LHD group [*p *> .05].

### Impact of memory task on priming performance

In order to determine if the dual task memory condition impacted the priming effects, Pearson correlations were carried out between memory task accuracy and mean RT priming effects (UNR - related RT). Correlations between memory accuracy and priming effects in each condition did not reach significance [*p *> .05], indicating that there was no trade-off between memory accuracy and priming performance for either group.

### Accuracy analysis

The LMM revealed main effects for target visual field [*F *(1, 9512) = 66.133, *p *< .001], and condition [*F *(1, 9501) = 132.226, *p *< .001]. The LMM also revealed a two-way interaction between group and target visual field [*F *(1, 9512) = 7.316, *p *= .007]. Both groups exhibited an advantage for RVF/LH accuracy (Control: 91.9%; LHD: 81.8%) compared to the LVF/RH (Control: 84.2%; LHD: 78%) [LHD: *p *= .001; Control: *p <*.001]. The control group performing significantly better than the LHD group targets in the RVF (Control: 91.9%; LHD: 81.7%) [*p *= .003], and the group difference in the LVF just reached significance [*p *= .043] (Control: 84.2%; LHD: 78%). The LMM also revealed an interaction between target visual field and condition [*F *(3, 9501) = 11.884, *p *< .001], and three-way interactions between group, memory condition and visual field [*F *(2, 9501) = 4.819, *p *= .008], and between group, target visual field and condition [*F *(3, 9501) = 3.122, *p *= .025]. In order to explore these higher order interactions, the data were analyzed for each memory condition separately to explore group, condition and target visual field factors. Table 4 lists the mean error rates for both groups in each condition and each memory load.

**Table 4:** Estimated marginal means of proportion accuracy for LHD group and Control group participants

*Condition*	*0 word memory*				*2 word memory*				*6 word memory*			
	
	*LHD*		*Control*		*LHD*		*Control*		*LHD*		*Control*	
	
	*LVF*	*RVF*	*LVF*	*RVF*	*LVF*	*RVF*	*LVF*	*RVF*	*LVF*	*RVF*	*LVF*	*RVF*
CA	.903	.957	.951	.950	.885	.854	.927	.971	.893	.837	.977	.974
	
	(.038)	(.040)	(.030)	(.030)	(.038)	(.039)	(.031)	(.031)	(.038)	(.039)	(.031)	(.031)

CO	.736	.846	.839	.931	.811	.768	.830	.931	.785	.786	.859	.929
	
	(.038)	(.039)	(.030)	(.030)	(.038)	(.039)	(.031)	(.031)	(.037)	(.039)	(.031)	(.030)

AO	.761	.917	.859	.954	.801	.895	.853	.945	.737	.880	.836	.972
	
	(.038)	(.039)	(.030)	(.030)	(.038)	(.040)	(.031)	(.031)	(.038)	(.039)	(.031)	(.031)

UNR	.687	.711	.744	.814	.701	.658	.759	.818	.660	.707	.670	.838
	
	(.038)	(.039)	(.030)	(.031)	(.038)	(.039)	(.031)	(.031)	(.037)	(.039)	(.031)	(.031)

Estimated marginal means proportion correct, with standard error in parentheses. Covariates for the estimated marginal means are Target Duration and Repetition (as per LMM analysis). *Abbreviations: CA *Category Association, *CO *Category Only, *AO *Association Only, *UNR *Unrelated, *LHD *Left Hemisphere Damage group, *Control *Control group, *LVF *Left visual field, *RVF *Right visual field

### Accuracy analysis by memory load

**Figure 4:**
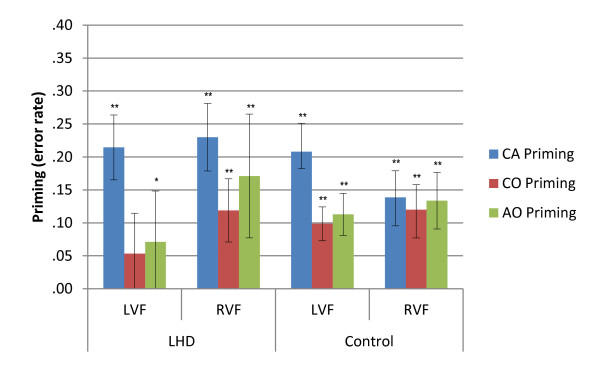
**Priming effects for error rate for LHD and Control groups in the zero-word memory condition**. Illustrated priming effects represent the mean of individual priming effects (Unrelated - Related condition error rate) in each condition. Error bars represent +/- 1 Standard Error. LHD = Left hemisphere damage; CA = Category Association; CO = Category only; AO = Association only; UNR = Unrelated; LVF = Left visual field; RVF = Right visual field; * p ≤ .05; ** *p *≤ .001.

The zero word memory condition accuracy data were analyzed using a LMM with group (LHD, Control), target visual field (LVF, RVF) and stimuli condition (CA, CO, AO, UNR) as factors, and target duration and target repetition as covariates. The LMM revealed a main effect for target visual field [*F *(1, 3219) = 38.245, *p *< .001], with both groups exhibiting RVF advantage for accuracy (LHD: 85.8%; Control: 91.8%) compared to the LVF (LHD: 77%; Control: 85.1%) [*p *< .001]. The LMM also revealed a main effect for stimuli condition [*F *(3, 3205) = 47.413, *p *< .001], and an interaction between target visual field and stimuli condition [*F *(3, 3205) = 3.807, *p *= .010]. In order to explore the pattern improvement between each of the related conditions and unrelated conditions, planned contrasts were carried out for both groups for each condition and in each visual field. Differences in error rate between related and unrelated conditions (priming) are illustrated in Figure 4. The contrasts revealed that the control group exhibited significantly more accurate performances in the CA [LVF and RVF: *p *< .001], CO [LVF: *p *= .001; RVF: *p *< .001], and AO [LVF and RVF: *p *< .001] conditions bilaterally, compared to the UNR condition. The LHD group exhibited better performance bilaterally for CA [LVF and RVF: *p *< .001] and AO [LVF: *p *= .048; RVF: *p *< .001] conditions, compared to UNR conditions. The CO condition was significantly different from UNR in the RVF only [*p *= .001] for the LHD group.

Priming magnitude for accuracy data (Unrelated error rate - Related error rate) was compared between conditions and visual fields using Wilcoxon Signed Ranks tests. Tests for the LHD group revealed a significantly greater priming effect for the CA condition (21%) compared to the CO (5%) [*z = -*2.547; *p = *.011] and AO (7%) [*z = -*2.310; *p = *.021] conditions in the LVF. In the RVF, the CA condition (23%) had a significantly greater priming effect than the CO condition (12%) [*z = -*2.310; *p = *.021]. The control group also exhibited a greater error rate priming for the LVF CA condition (21%) compared to both the CO (10%) [*z = -*2.760; *p = *.006] and AO conditions. (11%) [*z = -*2.970; *p = *.003]. No other comparisons reached significance.

**Figure 5:**
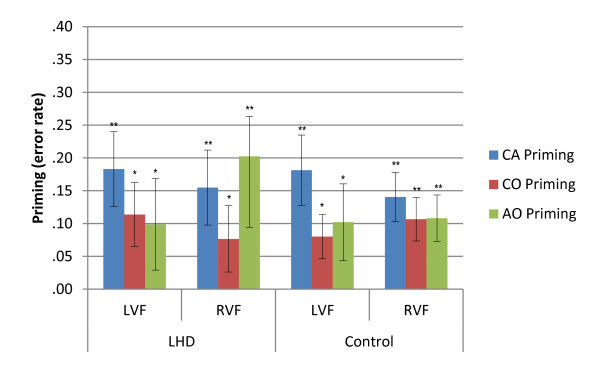
**Priming effects for error rate for the LHD and Control groups in the two-word memory condition**. Illustrated priming effects represent the mean of individual priming effects (Unrelated - Related condition error rate) in each condition. Error bars represent +/- 1 Standard Error. LHD = Left hemisphere damage; CA = Category Association; CO = Category only; AO = Association only; UNR = Unrelated; LVF = Left visual field; RVF = Right visual field; * p ≤ .05; ** *p *≤ .001.

The LMM for the two-word memory condition accuracy data revealed significant main effects for target visual field [*F *(1, 3112) = 10.507, *p *= .001], with the RVF (86.0%) advantage for accuracy compared to the LVF (82.3%). The LMM also revealed a main effect for stimuli condition [*F *(3, 3101) = 38.246, *p *< .001], as well as significant interactions between group and target visual field [*F *(1, 3112) = 7.991, *p *= .005], and between target visual field and condition [*F *(3, 3101) = 2.726, *p *= .043]. Group differences for target visual field performance were explored using planned contrasts between the visual field accuracy data for each group separately. The contrasts revealed a difference between the groups for RVF performance, with the control group exhibiting significantly more accurate responses (92.2%) than the LHD group (79.7%) [*p *= .016]. A hemispheric difference was revealed for the control group, with the RVF targets responded to more accurately (92.2%) than the LVF targets (84.9%) [*p *< .001]. The difference between visual fields did not reach significance for the LHD group [*p *= .830]. Planned contrasts were then carried out for each group between the conditions in each visual field to explore the pattern of performance for each condition at this memory condition. The planned contrasts revealed significant bilateral difference from UNR for each of the related conditions for both the control group; CA [LVF and RVF: *p *< .001], CO [LVF: *p *= .017; RVF: *p *< .001], and AO [LVF: *p *= .002; RVF: *p *< .001], and the LHD group; CA [LVF and RVF: *p *< .001], CO [LVF: *p *= .003; RVF: *p *= .006], and AO [LVF: *p *= .005; RVF: *p *< .001]. Mean difference in error rates are illustrated in Figure 5.

In the two-word memory load condition, the comparisons for the LHD group found a significant difference in accuracy error rate priming effect between the CA (18%) and AO (10%) conditions in the LVF [*z = -*2.100; *p = *.036] and between the CO (8%) and AO (20%) conditions in the RVF [*z = -*2.103; *p = *.035]. The LHD group had a RVF advantage (20%) for priming the AO condition compared to the LVF (10%) [*z = -*2.310; *p = *.021]. The control group comparisons revealed a significantly greater priming effect for the CA condition (18%) compared to both the CO (8%) [*z = -*2.669; *p = *.008] and AO conditions (10%) [*z = -*2.481; *p = *.013] in the LVF.

**Figure 6:**
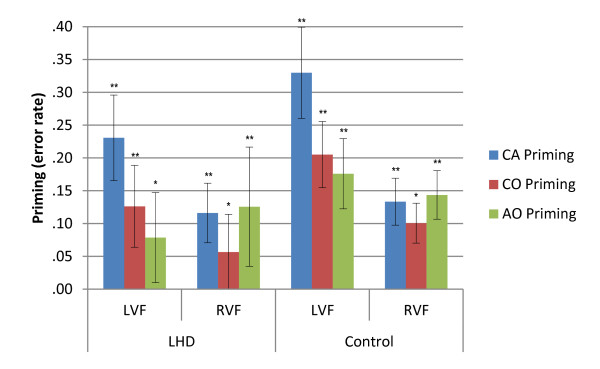
**Priming effects for error rate for LHD and Control groups in the six-word memory condition**. Illustrated priming effects represent the mean of individual priming effects (Unrelated - Related condition error rate) in each condition. Error bars represent +/- 1 Standard Error. LHD = Left hemisphere damage; CA = Category Association; CO = Category only; AO = Association only; UNR = Unrelated; LVF = Left visual field; RVF = Right visual field; * p ≤ .05; ** *p *≤ .00.

The LMM for the six word memory condition accuracy data revealed significant main effects for the factors target visual field [*F *(1, 3157) = 24.876, *p *< .001], and stimuli condition [*F *(3, 3150) = 48.3, *p *< .001], and significant interactions between target visual field and condition [*F *(3, 3150) = 9.713, *p *< .001], and between group and target visual field [*F *(1, 3157) = 6.331, *p *= .012]. The target visual field main effect reflected an overall accuracy advantage for the RVF (87.9%) compared to the LVF (80.4%). Group differences in hemispheric performance were explored using planned contrasts between visual fields for each group separately. The contrasts revealed a significant difference between the groups for RVF/LH performance, with the control group (93.6%) exhibiting significantly more accurate responses than the LHD group (80.3%) [*p *= .011]. There was also a significant hemispheric difference revealed within the control group, with the RVF targets responses (93.6%) being significantly more accurate than the LVF targets (84.2%) [*p *< .001]. The hemispheric difference did not reach significance for the LHD group [*p *= .099]. The condition by target visual field interaction was explored for each group via planned contrasts between the conditions for each visual field, and the difference in accuracy performance are illustrated in Figure 6. The contrasts revealed significant bilateral differences from UNR for both groups in each of the related conditions; CA [LHD: LVF *p *< .001, RVF *p *= .001; Control: LVF and RVF *p *< .001], CO [LHD: LVF *p *= .001, RVF *p *= .044, Control: LVF *p *< .001, RVF *p *= .003], and AO [LHD: LVF *p *= .032, RVF *p *< .001, Control: LVF and RVF *p *< .001].

Comparisons of magnitude of improvement in accuracy (between unrelated and related targets) for the LHD group found a significant difference in the error rate priming effect between the CA condition (23%) and both the AO (8%) [*z = -*2.666; *p = *.008] and CO (12.6%) [*z = -*2.073; *p = *.038] conditions in the LVF, and a LVF advantage for the CA condition (23%) error rate priming effect, compared to the CA RVF (12%) [*z = -*2.192; *p = *.028]. The control group comparisons revealed a significantly greater priming effect for the CA condition (33%) compared to the CO (21%) [*z = -*3.296; *p = *.001] and the AO (18%) [*z = -*3.180; *p = *.001] conditions in the LVF, and a significant difference between priming effects for the CO (10%) and AO (14%) conditions in the RVF [*z = -*2.100; *p = *.036]. Comparisons of conditions between visual fields revealed a LVF advantage for both the CA (LVF: 33%; RVF: 13%) [*z = -*2.982; *p = *.003] and CO conditions (LVF: 21%; RVF: 10%) [*z = -*2.605; *p = *.009], compared to the RVF for the control group.

## Discussion

The current investigation combined the dual task and DVF paradigms in order to explore the application of the RH disinhibition hypothesis to language processing following a LH lesion. In the current investigation, RH priming performance for individuals in the LHD group differed between the single task and dual task conditions, with increased RH priming found following the introduction of the concurrent memory loads. A similar change in performance was not evident for participants in the control group, with bilateral priming observed for all related conditions throughout the zero-, two-, and six-word memory load conditions.

### Priming performance in healthy aging

During the zero-word memory load condition it is possible to observe the baseline hemispheric semantic priming performance for the control group. In accordance with past investigations of young healthy controls, it is expected that the category association condition will exhibit bilateral activation [e.g., [18,20]], while category only stimuli can be expected to prime in the RH under post-lexical priming conditions [26-28], and the association only stimuli are expected to show LH priming at 250 ms SOA [e.g., [22]]. The bilateral priming pattern found for the current control group differs from the expected unilateral priming for the category only and association only conditions.

The control group's pattern of bilateral priming in all of the related conditions may be the result of the current investigations use of central primes and controlled post-lexical priming conditions and/or because of the age difference between the participants in the current investigation and the young healthy controls involved in previous investigations. The use of a central prime in DVF investigations has been associated with bilateral priming [e.g., [19,20]], particularly under controlled priming conditions. This is suggested to be because of a bilateral spread of semantic activation following the presentation of the prime. The incorporation of a central prime was important in the current investigation, as it provides a more realistic estimation of hemispheric contributions under normal reading conditions, compared to lateralized prime presentations [17].

Another possible explanation for the bilateral pattern of priming observed for the control group is that it is the result of to an age related reduction in hemispheric asymmetry for semantic processing. The Hemispheric Asymmetry Reduction in Older adults (HAROLD) model suggests that healthy older people experience a reduction in lateralization of processing on a range of cognitive tasks, compared to young healthy controls [58]. This is based on consistent findings of bilateral hemispheric activation of the pre-frontal cortex for older healthy adults, compared to lateralized activation in young healthy controls when performing the same tasks [see [58]]. Recent evidence extends the applicability of the HAROLD model specifically to behavioral semantic priming, with findings of a bilateral priming pattern for older controls and unilateral priming for young controls on an affective priming task [59]. Therefore, the bilateral pattern of priming found in the current investigation, compared to unilateral activation in previous young healthy control investigations [26-28] could reflect an age related compensatory mechanism of bilateral recruitment during semantic processing. This explanation would be strengthened by a direct comparison with a young healthy control group.

### Healthy aging and inhibitory control

The control group's pattern of bilateral priming in all of the related conditions continued into the two- and six-word memory load conditions. The consistency of RH priming performance, as the memory load increased, may indicate that neither the two- nor the six-word memory loads was sufficient to overload the healthy LH's attentional capacity and disinhibit RH processing. However, changes evident in hemispheric processing advantages across memory load conditions and hypotheses of age related decline in working memory suggest that a lack of LH overload may not be the best explanation.

The verbal memory load is designed to tax the resources of the LH and remove its processing advantage. Aging is widely accepted to reduce a person's working memory capacity [see [60,61] for reviews] due to a reduction in processing capacity [e.g., [60,62]], or a decline in the ability to inhibit activation of irrelevant information [e.g., [63-65]]. This deficit should increase the likelihood that older healthy adults would be more susceptible to the interference of the secondary memory load conditions, compared to young healthy adults. In previous investigations, a six-word verbal memory load has been sufficient to overload the LH's attentional resources and remove the no-load condition RVF/LH advantage for young healthy controls [6,66]. In contrast, the current control participants did not exhibit the expected RVF/LH RT advantage in the zero-, two- or six-word conditions. The lack of hemispheric advantage can be interpreted to reflect the age related reduction in processing asymmetry, compared to young healthy control participants.

A further hemispheric difference was evident for the control group at the zero-word memory load condition, with comparisons of priming magnitude revealing a RH advantage for priming the association only condition. DVF investigations with young healthy participants suggest that the association only condition should elicit priming in the LH only [e.g., [22]]. While bilateral activation may occur because of the central prime, the RH advantage cannot be similarly explained. The current investigation hypothesized that priming of the association only condition in the RH would provide an indication of RH disinhibition. Accordingly, the observed RH advantage for this condition reflects RH performance that exceeds the expected performance at the zero-word memory load condition.

Taken together, the advantage for RH priming performance in the association only condition, and the lack of a RVF/LH RT advantage overall, suggest a reduction of both LH dominance and RH inhibition. However, this "disinhibited" processing is occurring not only under memory load conditions, but also prior to the introduction of any memory loads. The occurrence of disinhibition in the no-load condition may also be related to the age of the current participants. Previous studies have found that healthy aging can result in a similar cognitive performance and similar pattern of PET brain activity to that observed for young healthy adults when their attentional resources are reduced by a divided attention task [e.g., [67]]. Similarly, the resource perspective of cognitive aging [e.g., [62,68]] suggests that aging reduces the supply of attentional resources available to the dominant hemisphere for processing. If this is the case, a memory load condition may not be necessary to facilitate the recruitment of additional processing resources from the non-dominant hemisphere in healthy older adults. Consistent with the HAROLD model, it is plausible that older controls will need to recruit more neural areas, such as the non-dominant hemisphere, in order to have access to an equivalent supply of attentional resources as young healthy controls and maintain similar performance accuracy [69]. In order to access resources from the non-dominant hemisphere it will need to be released from interhemispheric inhibition, similar to the modification of hemispheric attentional balance that underlies RH disinhibition within the dual task paradigm [2,5].

The benefit of the bilateral recruitment mechanism is suggested to be the maintenance of performance accuracy, despite the cognitive limitations of aging. In the current investigation there was a LH accuracy advantage evident in all three of the memory load conditions. This may reflect the compensatory nature of the bilateral processing, which serves to maintain performance accuracy comparable with younger controls [58]. However direct comparison with younger participants would be able to better verify and determine the nature of this potential compensatory mechanism.

The control group in the current investigation does not appear to require the secondary memory task in order to overload the LH and release the RH from inhibition, with evidence suggesting disinhibition of RH processing in the no-load condition. The similarities between the patterns of priming performance over the three memory load conditions are therefore speculated to result from a continuation in the strategic reduction in interhemispheric inhibition to continue to facilitate compensatory bilateral recruitment in older adults in each of the memory load conditions.

### Hemispheric semantic and associative priming following LHD

The LHD group exhibited hemispheric differences in priming performance during the zero-word memory load condition, with LH priming observed for all related conditions and RH priming for the category and association, and the association only conditions, but not the category only condition. To the authors' knowledge there are no previous relevant behavioral DVF investigations of semantic and/or association priming with people following LH lesions with which to compare the current hemispheric performances. Traditional priming studies, with a centrally presented prime and target, have found that people with LH lesions are able to prime items related via association and/or semantic category membership under automatic priming conditions [e.g., [70,71]].

Bilateral activation of the category association condition reflects findings from investigations of young healthy participants [e.g., [20]], and from the current investigation's control group. However, bilateral priming for the association only condition and the lack of priming for the category only condition in the RH do not reflect the a priori hypotheses of the current investigation. Bilateral activation in the association only condition does, however, mirror the performance of the control group in the current experiment, is consistent with hypotheses of bilateral activation for older participants [58], and fits with previous central prime DVF investigations [23].

The pattern of LH but not RH priming for the category only condition was not expected in the current investigation. According to the time-course hypothesis, however, priming of category only targets can be found unilaterally in the LH at particularly short SOAs (e.g., 150 ms) and under automatic processing conditions [16]. Activation in the LH only for the category only condition at 250 ms SOA may therefore reflect an increase in the time it takes for the damaged LH to focus activation on strongly related items, or may reflect a more general reduction in the ability to focus activation. Current results from priming magnitude comparisons in the LH reflect broad unfocused activation, with no significant differences found between the three related conditions. An advantage for the category and association condition over the other related conditions is expected for the LH, with this broad activation pattern associated with the RH in healthy controls [72]. In contrast, current findings suggest a RH advantage for priming targets with an association relationship, with both the category membership and association, and association only conditions exhibiting greater priming magnitude than the category only condition. This suggests that following a LH lesion the RH is benefiting from an association boost in the zero-word memory load condition. An association boost refers to the reduction in processing required to activate associated stimuli, therefore making them easier to process, compared to the weaker category only relationship [21]. In older individuals with lesions, it is assumed that there would be limited processing resources compared to young healthy controls. The resource limitations, in combination with interhemispheric suppression, would result in the RH being unable to perform to its full processing capacity. Therefore, it is hypothesized that the RH would only have sufficient processing resources to activate associated items. Overall, it appears that following a LH lesion, hemispheric no-load priming patterns reflect a broad spread of activation in the LH and processing in the RH appears to require an association boost in order to activate, potentially due to interhemispheric inhibition and resource limitations.

### Disinhibition following a LH lesion

The current investigation aimed to directly explore suggestions that a LH lesion will result in RH disinhibition [30,31], by experimentally manipulating RH disinhibition for these participants. Individuals in the LHD group exhibited an increase in RH participation under dual task conditions. At baseline (no-load condition) the LHD group exhibited an overall LH advantage for processing and no priming for the category only condition in the RH. Following the introduction of a verbal memory load the LHD group were found to significantly prime all related conditions in the RH, to exhibit a priming magnitude advantage in the RH for the category association condition (two-word memory load), and no longer exhibited the LH processing advantage that had been present at baseline. As in previous investigations of healthy controls [e.g., [6,73]], the removal of LH processing advantage under dual task conditions indicates that the additional verbal memory task was successful in overloading the attentional resources of the LH, and resulted in disinhibition of RH processing.

The impact of verbal memory load on LHD group processing is in contrast to findings of the current control group, particularly as it indicates RH inhibition in the no load condition. A LH lesion will result in the reorganization of hemispheric contributions to language processing, and the possible use of different compensatory mechanisms. These mechanisms are discussed in the remainder of this section. The LHD group findings also differ from previous investigations that have found evidence of RH disinhibition following LH lesions [30,31], and from investigations that have found RH disinhibition following simulated rTMS lesions in the LH [11].

The current cohort of participants differed from LHD participants in previous investigations, who either had significantly large lesions [31], severe language deficits [30], or 'acute' simulated lesions [11]. Lesion size is suggested to influence disinhibition, with Landis et al. [31] suggesting that RH disinhibition following a LH lesion requires the complete isolation of the RH, and that this most likely occurs only following particularly large lesions. Landis et al. [31] went on to speculate that if a lesion was not significantly large enough, the dominant LH may maintain inhibition of RH language processing, as may be the case for the current LHD group.

Another possible explanation for the lack of spontaneous RH disinhibition for LHD participants in the current investigation is that the extent of spontaneous disinhibition is relative to either the degree of continuing language impairment. The current participants were considered to exhibit high levels of residual or recovered language abilities, with current classifications of either mild residual aphasia or no residual aphasia on the WAB [41]. This average profile compares with the participant discussed by Landis and Regard [30] who continued to experience severe global aphasia at 1 year post lesion and demonstrated continuing evidence of RH disinhibition. The extent of LH inhibition may be related to the extent of continuing language impairment, either because a lack of recovery has not allowed the LH to regain inhibitory control, or because successful recovery results from the reintroduction of LH control.

Hierarchical theories of language reorganization and recovery following LH lesion have combined these arguments somewhat, by suggesting that success of recovery may be linked to an interaction between lesion size, damage to key functional networks and the subsequent degree of intra- and interhemispheric disinhibition [8]. Specifically, Heiss and Theil [8] suggest that the most successful recovery of language ability is associated with the restoration of function in the original language processing regions of the LH, and that this is only possible for particularly small lesions. Following that, intrahemispheric compensation can be employed following damage to key LH language areas, via the reduction of intrahemispheric inhibition within the dominant hemisphere. This reorganization is suggested to encourage potentially incomplete, but satisfactory recovery. Finally, severe damage to a large region of LH networks is suggested to reduce interhemispheric inhibition and encourage reorganization to homologous RH language regions, which is associated with the least successful language recovery [8].

Alternatively, the extent of interhemispheric disinhibition following a unilateral lesion may relate to the amount of time elapsed since lesion. This may explain the differences in findings between investigations of rTMS induced lesions, which indicate the potential for RH disinhibition in the acute stages of a simulated lesion and the findings of the current investigation, whose participants are in the chronic stages with at least 10 months since lesion onset. There is the potential therefore that RH disinhibition post lesion may be short lived, with a return of LH control in the chronic phases. Findings from a series of longitudinal single case studies by Ansaldo and colleagues [13-15,74] provide support for the relationship between recovery time-course and the degree of RH disinhibition observed, although disinhibition was not the focus of the investigation or explanation provided by Ansaldo and colleagues. Ansaldo and Arguin [13] described an initial advantage for RH processing, with an extension of processing abilities beyond those associated with RH processing in a non-lesioned brain. The extension of processing abilities in the RH at this early stage may reflect an initial release from LH inhibition. Following the initial RH advantage, an improvement of LH function was observed, and the hemispheres appeared to perform equivalently at one year post lesion. The improvements observed in LH functioning as recovery progressed [13], in combination with the findings of reduced RH processing abilities for the participants in the LHD group during the single task condition in the current investigation, may suggest that following sufficient time post lesion or sufficient degree of recovery the LH is able to regain its control of RH processing. The suggested changes to RH disinhibition over time following LH lesion is also supported by findings from neuroimaging investigations that suggest an upregulation of RH homologues for language processing during the acute/subacute phases, that is followed, under certain circumstances (depending on lesion size etc.), by language processing being carried out in ipsilesional LH regions coupled with a return of LH control [e.g., [75]]. The LHD participants in the current study are in the chronic stage post lesion, and the observed inhibition of the RH is speculated to be important to facilitate the reintroduction of LH dominance for language processing over time.

### Limitations and future directions

Methodological limitations may have influenced the priming patterns that were found in the current investigation. In particular, the use of individualized presentation times for lateralized stimuli, that exceeded 200 ms in some cases, may have increased the likelihood of eye movement and therefore access to a target via both visual fields, although the likelihood of this occurring was minimized with monitoring. In addition, due to the nature of the task there was a considerable amount of data lost due to observed eye movement and a high rate of error, particularly for the LHD group. This may have influenced the RT results reported. The current methodology did not counterbalance the response hand for the lexical decision task between participants. This is an issue for two reasons. Firstly, because the majority of participants responded with their right hand and this may have caused an advantage for LH processing. Secondly, because the use of the post-morbid dominant left hand for some LHD patients may have similarly caused a RH advantage in that group. Finally, interpretation of results may have benefited from the inclusion of more specific lesion information, such as etiology and lesion size, for each of the individuals in the LHD group.

Future investigation of RH disinhibition would benefit from comparing a larger cohort of individuals with varying lesion sizes, in order to directly explore the effect of particular lesion characteristics on RH disinhibition. Future investigations should also consider case-by-case analysis to provide insight into differences in disinhibition between individuals. Measuring language recovery over time (from acute to at least 1 year post lesion) in combination with the experimental manipulation of disinhibition, would allow more direct examination of RH disinhibition over time and language recovery. Finally, it would be interesting and theoretically relevant to directly compare older and younger controls on tasks that manipulate hemispheric inhibition in order to learn more about the impact of aging on interhemispheric control.

## Conclusions

Overall, the current investigation provides evidence that RH contributions to semantic processing following LH lesions can be manipulated and enhanced by introducing a secondary task that engages the processing resources of the LH. However, for these participants the LH lesion alone did not appear to result in RH disinhibition at the time of testing. This finding has implications for theories of hemispheric inhibition following lesions and over recovery. Investigations that directly explore language recovery and incorporate a dual task paradigm with participants following LH lesions have the potential to expand the current knowledge regarding the dynamic interaction between the hemispheres during language processing and recovery following unilateral LH lesions.

## Competing interests

The authors declare that they have no competing interests.

## Authors' contributions

ES-C participated in the design of the study, carried out data collection, completed the data analysis and interpretation, and wrote the manuscript. HC participated in the design of the study and in manuscript preparation and editing. AA contributed to the data analysis, interpretation of the results, and manuscript drafting and editing. DC was senior author, contributing to the design of the study, data analysis and interpretation, and manuscript drafting and editing. All authors read and approved the final manuscript.
